# Disruption of Rolandic Gamma-Band Functional Connectivity by Seizures is Associated with Motor Impairments in Children with Epilepsy

**DOI:** 10.1371/journal.pone.0039326

**Published:** 2012-06-21

**Authors:** George M. Ibrahim, Tomoyuki Akiyama, Ayako Ochi, Hiroshi Otsubo, Mary Lou Smith, Margot J. Taylor, Elizabeth Donner, James T. Rutka, O. Carter Snead, Sam M. Doesburg

**Affiliations:** 1 Division of Neurosurgery, Hospital for Sick Children, Toronto, Ontario; 2 Division of Neurology, Hospital for Sick Children, Toronto, Ontario; 3 Institute of Medical Science, University of Toronto, Toronto, Ontario; 4 Department of Medical Imaging, University of Toronto, Toronto, Ontario; 5 Neurosciences and Mental Health Program, Hospital for Sick Children Research Institute, Toronto, Ontario; 6 Department of Psychology, University of Toronto, Toronto, Ontario; 7 Department of Diagnostic Imaging, Hospital for Sick Children, Toronto, Ontario; Zhejiang University School of Medicine, China

## Abstract

Although children with epilepsy exhibit numerous neurological and cognitive deficits, the mechanisms underlying these impairments remain unclear. Synchronization of oscillatory neural activity in the gamma frequency range (>30 Hz) is purported to be a mechanism mediating functional integration within neuronal networks supporting cognition, perception and action. Here, we tested the hypothesis that seizure-induced alterations in gamma synchronization are associated with functional deficits. By calculating synchrony among electrodes and performing graph theoretical analysis, we assessed functional connectivity and local network structure of the hand motor area of children with focal epilepsy from intracranial electroencephalographic recordings. A local decrease in inter-electrode phase synchrony in the gamma bands during ictal periods, relative to interictal periods, within the motor cortex was strongly associated with clinical motor weakness. Gamma-band ictal desychronization was a stronger predictor of deficits than the presence of the seizure-onset zone or lesion within the motor cortex. There was a positive correlation between the magnitude of ictal desychronization and impairment of motor dexterity in the contralateral, but not ipsilateral hand. There was no association between ictal desynchronization within the hand motor area and non-motor deficits. This study uniquely demonstrates that seizure-induced disturbances in cortical functional connectivity are associated with network-specific neurological deficits.

## Introduction

Children with epilepsy are known to exhibit varying levels of neuropsychological impairments ranging from motor weakness to deficits in cognition, perception and memory [1], [2]. Prolonged refractory seizures are associated with increased functional impairment, and early control of epilepsy is imperative to counteract developmental deficits [3], [4]. Furthermore, it is common for patients with focal epileptogenic lesions to present with diffuse alterations of cognitive function, which cannot be exclusively attributed to the location of the lesion. Although recent research has suggested that performance difficulties associated with epilepsy may arise due to disruption of functional connectivity within distributed brain networks [5], [6], the mechanism of seizure-induced network impairment remains unclear.

Initially described in the visual cortex, oscillatory neural synchronization in the gamma frequency range (>30 Hz) is thought to dynamically modulate functional connectivity among neural populations [7], [8]. Synchronization of gamma oscillations is also understood to underlie coordination of activity within distributed task-dependent neuronal assemblies [9], [10] supporting numerous processes including sensory integration, attention, action selection as well as learning and response inhibition [11]. Oscillatory synchronization among brain regions has been implicated in motor control, and its disturbance has been studied in clinical populations [12]. Maturation of gamma oscillations is related to the development of cognition and perception during childhood and adolescence [13], [14], and atypical patterns of oscillatory coherence are associated with conditions affecting childhood cognitive development [15], [16].

Aberrant brain synchronization has long been thought to play a critical role in epileptic seizures [17], [18] and experimental observation has confirmed abnormal synchrony within epileptogenic brain regions [19], [20]. Recent application of graph theoretical analysis has also revealed that network properties of functional connectivity are abnormal in epileptic cortex and that these areas are functionally disconnected from other brain regions [19], [21], [22]. Despite convergent evidence implicating network connectivity in cognitive, perceptual and motor function, their impairments in clinical populations, and recent findings linking oscillatory power to functional difficulties in epilepsy [23], the relationship between oscillatory synchrony and functional deficits in epilepsy remains poorly understood.

The study uniquely investigates relationships among network synchrony and functional impairment in epilepsy. Specifically, we evaluate the consequences of uncontrolled epileptic seizures on motor networks by evaluating their impact on functional connectivity involving the Rolandic cortex. The identified network properties are compared with neuropsychological assessments to identify associations between network connectivity and clinical motor deficits. Furthermore, to evaluate whether these effects are due to relations among neural synchrony or rather a reflection of the location of the epileptogenic cortex, we include patients with Rolandic and extra-Rolandic epilepsy and adjust for the location of the intracranial electroencephalographic (iEEG) seizure-onset zone (SOZ) and/or the epileptogenic lesion on magnetic resonance imaging (MRI) relative to the Rolandic cortex.

## Methods

### Patient Population

We obtained iEEG recordings from fifteen children undergoing invasive monitoring for surgical treatment of medically-intractable focal epilepsy at the Hospital for Sick Children. The underlying pathology in all cases was focal cortical dysplasia (FCD), as classified by the International League Against Epilepsy [24]. The protocol for the analyses described herein was reviewed and approved by our The Hospital for Sick Children research ethics board. Retrospective electrophysiological data were reviewed and no prospective patient consent was required.

**Figure 1:**
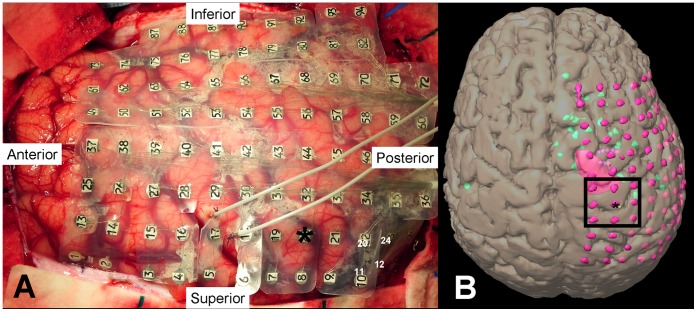
Intraoperative photograph showing placement of grid over right hemisphere. (A) Asterisk indicates motor hand area as determined by cortical stimulation. (B) Three-dimensional reconstruction showing grid (light pink dots), MRI lesion (dark pink area), magnetoencephalographic (MEG) cluster (green dots). Square shows 3×3 montage used for PLV analysis.

The mean age was 11.3±4.2 years with a mean duration of epilepsy of 5.3±3.1 years and a mean daily seizure frequency of 4.8±4.4 seizures per day. The majority of children (10 patients; 67%) had type II FCD with balloon cells visible on microscopic examination. Subdural grids were used (median 111 electrodes; range 98–122). A sample case is presented in **Figure 1**. There were no significant differences between children with normal and abnormal motor function with respect to duration of epilepsy (p = 0.35), seizure frequency (p = 0.15), number of distinct seizures (p = 0.56) or size of the SOZ (p = 0.17). The distance between the SOZ and the Rolandic cortex was, however, significantly less in children with abnormal motor function (p = 0.02). None of these children exhibited post-ictal (Todd’s) paresis.

The technique of subdural grid implantation and intra- and extra-operative functional mapping of the epileptogenic and eloquent cortices have been previously described [25]. We used subdural grids of 4-mm diameter electrodes embedded in a silicone elastomer sheet with interelectrode distances ranging from 8 to 10-mm. Patients underwent digitally recorded intracranial video-EEG using a Harmonie system (Stellate, Montreal, QC, Canada) with a sampling rate of 1 kHz and anti-aliasing filter at 300 Hz (Butterworth, −20 dB/oct) applied prior to sampling. An averaged reference was selected by clinical electrophysiologists from two channels in a relatively inactive area of the grid during seizures, which was also distant from Rolandic cortex. Children were included in the study if the subdural grid covered the motor cortex and the hand motor area was reliably identified by cortical stimulation.

Ictal and interictal epochs were selected based on iEEG tracings. Ictal periods were of variable length and were comprised of rhythmic iEEG activity demonstrating evolution over time and associated with clinical seizures. Interictal epochs were each two minutes in length and selected at least an hour apart from ictal events. The interictal periods were chosen by experienced electrophysiologists as representative background activity, which in most cases, included interictal epileptic discharges. The iEEG sections were exported as European Data Format Plus (EDF+) files [26] and imported into MATLAB software for subsequent analyses (The MathWorks, Natick, MA, USA). At least three epochs of each type were analyzed for each patient and the mean phase-locking and clustering values were calculated.

### Phase Synchronization Analysis

To determine synchronization within the motor cortex, we extracted a Rolandic region of interest (ROI) including data from the electrode determined by extra-operative stimulation to be over the hand motor area and its adjacent electrodes (e.g. a 3 by 3 electrode montage centred over the motor hand area). An internal control was chosen by defining another 3 by 3 montage of electrodes at least three electrodes away from the motor hand area and as equidistant as possible to the SOZ. To investigate long-range phase synchronization involving motor cortex, a secondary analysis was performed including all pairs of electrodes within the grid. The data were band-pass filtered digitally at 1 Hz intervals between 4 and 300 Hz with a notch filter applied to all resonance frequencies of 60 Hz to exclude line noise. The analytical signal of the filtered waveform for each ictal and interictal epoch *f(t)* was calculated to obtain the instantaneous phase 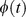, where 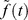
is the Hilbert transform of 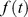:
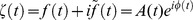
Inter-electrode phase synchronization was quantified using phase-locking values (PLVs). PLVs were calculated by comparing the instantaneous phases of signals recorded by pairs of electrodes across time [27]. As we hypothesized that seizures alter the functional connectivity of eloquent cortex, an average PLV value for each pair-wise electrode relation was derived for the entire course of each ictal and interictal epoch.

PLVs range between 0 (random phase difference) and 1 (maximum phase-locking). To determine inter-electrode synchrony within the motor cortex, the PLV values associated with all pair-wise comparisons of the electrodes within the motor ROI were averaged across each frequency band. The derived PLV was subsequently averaged across defined frequency bands: delta (1–4 Hz), theta (5–8 Hz), alpha (8–13 Hz), beta (14–30 Hz), gamma1 (36–44 Hz), gamma2 (45–80 Hz), gamma3 (81–150 Hz), HFO1 (151–200 Hz), HFO2 (201–250 Hz) and HFO3 (251–300 Hz). In order to quantify the disruption of functional connectivity associated with seizures, we subtracted the mean interictal phase-locking from ictal values within each analyzed frequency range, creating a composite variable describing the ictal phase desynchronization (hereafter referred to as ‘ictal desynchronization’).

### Graph Theoretical Analysis

To assess network connectivity involving motor cortex, we performed graph theoretical analysis using the Brain Connectivity Toolbox [28]. Each electrode on the grid was defined as a ‘node’ and the calculated PLV values represented the ‘edge’ weights, creating a weighted undirected network. A key measure of networks is the clustering coefficient [29]. This is defined by the ratio of the number of connections between the neighbours of a node and the number of all the possible connections between its neighbours [30]. By providing an indication of the embeddedness of a single node, clustering coefficients quantify the degree of connectivity within the synchronization network. We constructed our graphs using weighted undirected edges defined by PLVs and therefore derived a weighted clustering coefficient, representing the average ‘intensity’ of triangles around a node [31]. A frequency of 80 Hz was selected to investigate clustering in the gamma-band, as this was consistent with the frequency range which showed the strongest relationship between ictal desynchronization and motor function in the ROI analysis. Once the connectivity matrix of the entire grid was analyzed, all possible connections between the electrode recording from the motor cortex (the hand motor node) and the remainder of the electrodes (i.e. all functional neighbours) were considered in the analysis and the clustering coefficient was extracted [32]. As with the analysis of phase synchronization within Rolandic cortex, we subtracted the interictal clustering values from ictal values in order to index the seizure-induced disturbance of connectivity within functional systems involving motor cortex. This composite variable is termed ‘ictal declustering.’

### Neuropsychological Testing

The majority of children underwent a battery of neuropsychological testing including two motor-related tasks: the grooved pegboard (N = 10) [33] and finger tapping tasks (N = 9) [34]. Non-motor aspects of the children’s function were assessed with the matrix reasoning (N = 13) and vocabulary subsets (N = 12) of the Wechsler Intelligence Scale for Children [35]. Age- and gender-adjusted z-scores were derived [35], [36]. Children were dichotomized as having normal (N = 9) or abnormal (N = 6) motor function according to their neuropsychological evaluations with those performing under one standard deviation from the mean on the grooved pegboard task and/or with gross motor weakness of the contralateral hand on neurological examination. The grooved pegboard task was used to define abnormal hand function as it is the more complex motor task, requiring both speed and dexterity. All testing was administered and interpreted by neuropsychologists and neurologists during clinical care.

### Statistical Analysis

Data are presented as means with error bars representing the standard deviation unless stated otherwise. Binary and dichotomized categorical variables were analyzed using the two-tailed Fisher’s exact test. The means of continuous variables were compared using the nonparametric randomization test, which employs resampling techniques to yield exact significance levels [37]. To adjust for confounders, a one-way analysis of variance (ANOVA) or analysis of covariance (ANCOVA) was performed for categorical and continuous variables respectively. A multivariate logistic binary regression was also performed with variables selected for inclusion based on *a priori* hypotheses. Outcomes were considered statistically significant at a p<0.05. Statistical analysis was performed using SAS Statistical Software 9.3 (Cary, North Carolina).

## Results

### Ictal Gamma-band Desynchronization and Motor Impairment

**Figure 2:**
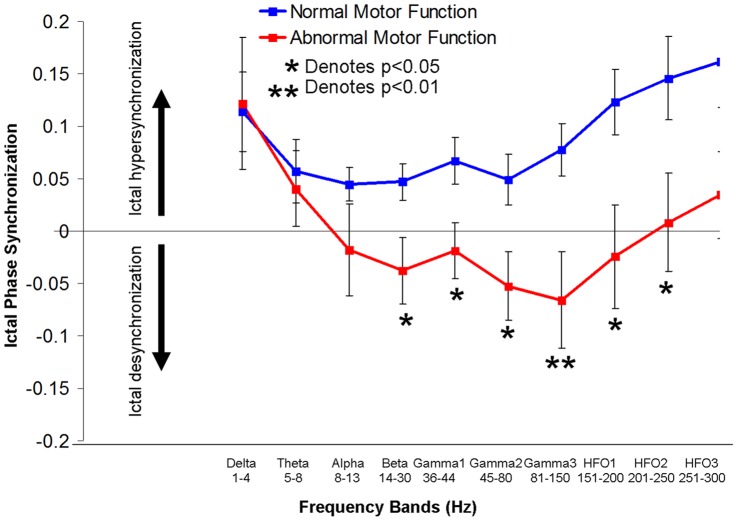
Differences in Rolandic phase-locking between ictal and interictal epochs (i.e. ictal phase synchronization) between children with normal and abnormal motor function across defined frequency bands. Children with motor deficits had ictal desychronization (relative to interictal period), most significantly in the gamma3 (81–150 Hz) band.

There was a trend towards a significant difference between ictal PLVs in the Rolandic ROI (3 by 3 electrode montage) in children with normal and abnormal motor function, most notably in the gamma2 (p = 0.053) and gamma3 (p = 0.06) frequencies. When we tested our composite variable, ‘ictal desynchronization’ – which has the notable advantage of accounting for baseline (interictal) differences in Rolandic connectivity between subjects – those with motor deficits were significantly more likely to have ictal desynchronization within the contralateral Rolandic ROI than those without deficit. This was observed across numerous frequency bands and was most significantly expressed in the gamma3 band (81–150 Hz: p<0.01; **Figure 2**). Because this frequency band yielded the greatest difference, it was selected for comparison with neuropsychological testing.

**Figure 3:**
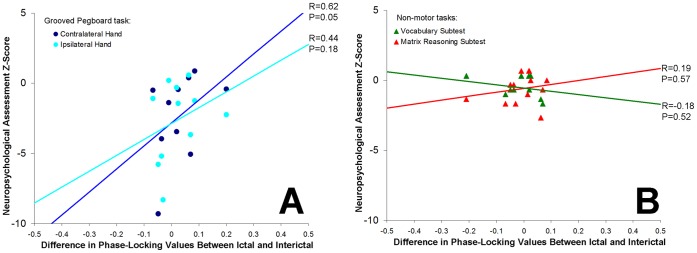
Linear regression of (A) motor tasks and (B) non-motor tasks with differences in phase-locking between ictal and interictal epochs. Extent of ictal desynchronization (relative to interictal epochs) was significantly correlated with degree of neuropsychological impairment for the grooved pegboard task in the hand contralateral to recording, but not the ipsilateral hand or non-motor tasks.

Performance on the grooved pegboard task of fine motor dexterity of the hand contralateral to recording showed a strong linear correlation with extent of ictal desynchronization at the gamma3 band (Pearson coefficient: 0.62; p = 0.05; **Figure 3**). The correlation of ictal desynchronization in the gamma3 frequency band with fine motor dexterity outcomes for the hand ipsilateral to recording showed a weaker and non-significant trend (Pearson coefficient: 0.44; p = 0.18) and correlation with non-motor neuropsychological function, the verbal and matrix reasoning subtests of WISC-IV showed no association (Pearson coefficients −0.18 and 0.19 respectively; p = 0.57 and 0.52 respectively). There was no significant correlation between ictal desynchronization and the tapping task in the contralateral or ipsilateral hands (Pearson correlations: 0.41 and 0.02; p = 0.27 and 0.96 respectively).

When adjusting for the presence of the iEEG defined seizure-onset zone (SOZ) and/or MRI lesion within the motor cortex using a two-way ANOVA, we found that significant differences in ictal desynchronization in the gamma3 frequency band (81–150 Hz) which exhibited the most significant association with motor function remained between children with normal and abnormal contralateral hand function (F-value = 6.48; p = 0.03). Furthermore, there was no interaction between ictal desynchronization in this frequency band and the presence of iEEG defined SOZ and/or MRI lesion (F-value = 0.73; p = 0.41). We included the frequency yielding the strongest difference between normal and abnormal motor function (gamma3, 81–150 Hz) in a multivariate logistic regression model and found that gamma-range ictal desynchronization in that frequency band was a stronger independent predictor of motor deficit (OR 29.97; 95% CI 1.41–637.67) than the presence of the iEEG-defined SOZ or MRI lesion within the motor cortex (OR 3.45; 95% CI 0.11–112.61).

When we adjusted for epilepsy duration using ANCOVA, we found that the association between ictal desynchronization at the 81–150 Hz band and abnormal contralateral motor function remained significant (F-value = 13.79; p<0.01). Longer duration of epilepsy (greater than 6 years) was also independently associated with worse neuropsychological outcomes on the grooved pegboard task (F-value 8.70; p = 0.03), but not the finger-tapping task (F-value = 0.86; p = 0.40). There was no significant interaction between ictal desychronization and epilepsy duration (F-value = 3.12 and 0.29; p = 0.18 and 0.61 on grooved pegboard and finger-tapping tasks respectively).

In our internal control montage, located at least three electrodes away from the hand motor area and equidistant from the SOZ, there was no difference in ictal desynchronization between children with normal and abnormal motor function at any frequency band (delta p = 0.38; theta p = 0.10; alpha p = 0.09; beta p = 0.29; gamma1 p = 0.25; gamma2 p = 0.29; gamma3 p = 0.64; HFO1 p = 0.80; HFO2 p = 0.78; and HFO3 p = 0.96). Furthermore, there was no significant correlation between ictal desynchronization at the gamma3 frequency band of the control montage and any neuropsychological test (data not shown).

### Graph Theoretical Analysis

**Figure 4:**
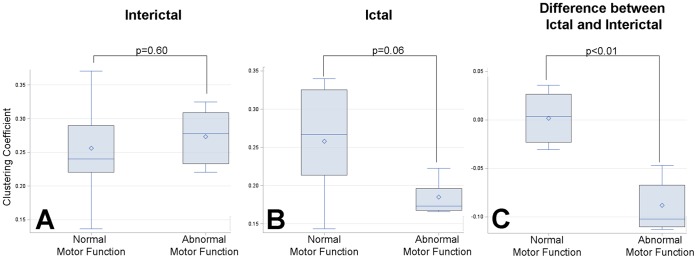
Graph theoretical analysis-based topographic mapping of grid showed significant local declustering in the Rolandic cortex during the ictal period relative to interictal epoch (ictal minus interictal) at 80 Hz in children with abnormal motor function. There was no significant difference between the two groups of children interictally and there was a trend towards more declustering in the ictal period (p = 0.06). There was a significant difference between the extent of ictal declustering (relative to interictal epochs) in children with normal and abnormal motor function.

**Figure 5:**
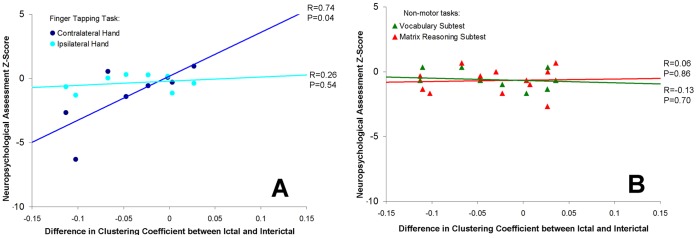
Linear regression of (A) motor tasks and (B) non-motor tasks with differences in clustering between ictal and interictal epochs. Extent of Rolandic ictal declustering (relative to interictal epochs) significantly correlated with the degree of motor impairment based on the Finger tap test in the contralateral, but not ipilateral hand and did not show significant correlation with non-motor deficits.

Analysis of graph theoretical properties of Rolandic cortex connectivity involving the entire electrode grid revealed that patients with hand motor deficits had significantly higher ictal declustering at 80 Hz involving the hand motor area of the contralateral Rolandic cortex compared relative to children without motor impairment, despite no significant differences in ictal and interictal clustering between these two groups (**Figure 4**). Of note, however, the trend towards significance during the ictal period (p = 0.06) suggests that seizures are driving the differences observed. Neuropsychological outcomes for the hand tapping task for the hand contralateral to recording showed a strong linear correlation with extent of ictal declustering at 80 Hz (Pearson coefficient: 0.74; p = 0.04; **Figure 5**). There was no significant association between the finger tapping of the ipsilateral hand, or non-motor tasks, and ictal declustering of gamma connectivity. For the grooved-pegboard test, there was a significant association between ictal declustering and outcome for the ipsilateral hand (Pearson coefficient: 0.66; p = 0.04), but not the contralateral hand (Pearson coefficient: 0.44; p = 0.23) or non-motor tasks.

After adjusting for the presence of the iEEG-defined SOZ and/or MRI lesion within the hand-motor area of the Rolandic cortex using a two-way ANOVA, the decrease in clustering coefficient at 80 Hz between the ictal and interictal period remained significantly different between children with normal and abnormal contralateral hand function (F-value = 6.23; p = 0.03). There was no significant interaction between iEEG-defined SOZ and/or MRI lesion location in Rolandic cortex and abnormal contralateral hand function (F-value = 0.02; p = 0.90). We also adjusted for epilepsy duration using ANCOVA and found that the observed differences in ictal declustering between children with normal and abnormal motor functions remained significant (F-value = 12.96; p<0.01). There was no significant interaction between duration of epilepsy and ictal declustering properties (F-value = 0.15; p = 0.30). In a multivariate logistic regression model, neither a binarized ictal declustering variable nor the presence of the SOZ/MRI lesion in Rolandic cortex was predictive of abnormal hand function.

## Discussion

The current study uniquely demonstrates that seizures alter the functional connectivity of eloquent cortical areas and that these alterations are predictive of clinical neurological deficit. We also provide direct evidence that seizure-induced alterations of connectivity in functional networks, which may be distant from iEEG-defined SOZ or presumptive epileptogenic MRI lesions, are associated with neurological impairments. We also provide the first evidence that invasion of function-specific areas of eloquent cortex by ictal connectivity dynamics are selectively related to impairment of the relevant functional domain (e.g. ictal desynchronization of motor cortex is selectively relevant to motor impairment). Hand motor function was selected to test this hypothesis as it represents a relatively simple, robust network, for which the implicated cortical regions are reliably identified through cortical stimulation [25]. Based on our findings, we speculate that ictal phase desynchronization and disruption of functional connectivity within a variety of distributed brain networks may underlie the broad spectrum of impairments affecting children with epilepsy. This view is consistent with EEG evidence linking gamma-band synchronization to the formation of distributed neuronal coalitions supporting a variety of cognitive and perceptual processes [38]–[42] as well findings of transient ictal desynchrony during aberrant emotional behaviour [43]. The observed association between ictal reduction gamma-band synchrony within Rolandic cortex and motor deficit may therefore represent a mechanism through which epileptic seizures exert long-lasting effects on cortical network dynamics and consequently neuropsychological function.

It remains unclear why in the present study seizure-induced changes in functional connectivity were found to be independently associated with motor weakness, although this finding is supported by clinical associations between longer duration of epilepsy and increased baseline functional impairment. One possible explanation is that prolonged desynchronization and disconnection of functional networks may facilitate network plasticity within the Rolandic cortex, resulting in clinical motor deficit. It has been previously shown that the capacity of individual neurons to exhibit adaptive changes or plasticity is influenced by gamma synchronization [44], [45]. Furthermore, coherence of oscillatory gamma-band EEG activity has been previously studied as a basis for cognitive processes necessitating neuronal plasticity, such as learning and memory [45]–[47]. A critical implication of our findings is that aberrant gamma synchrony may act to pathologically decorrelate neurons comprising a functional circuit, resulting in long-lasting disruptions in connectivity and thus motor impairments.

Another explanation for our findings involves the role of interictal discharges in disrupting networks beyond the ictal period, further contributing to network destabilization. Using EEG-correlated functional MRI, it has been reported that interictal epileptic discharges disrupt resting-state networks in a manner analogous to task performance [48]. However, it has also been shown that patients with epilepsy exhibit resting-state network impairments during interictal periods without interictal epileptic discharges and that functional connectivity is negatively correlated with disease duration [49]. Based on the findings of the current study, we hypothesize that dysfunctional network integration may be related to repeated ictal desychronization and functional disconnection within brain networks supporting motor function. This viewpoint is further buttressed by recent demonstration that phase correlations among gamma oscillations in distributed neuronal coalitions contribute to the formation of task specific network interactions involving the motor system [10].

A wealth of literature has also recently emerged suggesting that pathological high frequency oscillations (pHFOs; above 80 Hz) are a signature of cortical epileptogenicity [50], [51]. One study showed that the presence of ictal motor symptoms was related more to pHFO amplitude in Rolandic cortex than in the SOZ, and that augmentation of ripple-band pHFOs (80–200 Hz) occurred approximately 400 ms prior to EMG onset of ictal motor phenomenon [52]. In contrast to studying oscillation amplitude, we examined phase-locking synchrony and graph theoretical properties of gamma-band networks and showed that ictal disturbances in connectivity are associated with baseline motor deficit. Furthermore, we demonstrated that the magnitude of motor impairment was correlated with the extent of desynchronization and functional disconnection within Rolandic cortex. Using multivariate analysis, we have also shown that these findings are more predictive of deficit than epileptogenicity (SOZ presence) in the Rolandic cortex. It is also interesting that in the current study, the most significant frequency was the high-gamma band (81–150 Hz) (p<0.01), suggesting that ictal desychronization was strongest within the gamma-band, which has been reliably implicated in the formation of networks supporting cognition, perception and motor control. Finally, while previous studies have shown that the SOZ is itself functionally disconnected [19], we show that ictal disconnection of eloquent cortical areas independent of the SOZ location is associated with neurological deficits. We speculate that this may be a leading mechanism for neurological and cognitive impairment in children with epilepsy.

The present study possesses numerous advantages over previous works. Importantly, large subdural grids were used for electrocorticography, allowing us to capture a greater number of nodes and to more accurately apply graph theoretical analyses to map local and inter-regional connectivity involving Rolandic cortex. Secondly, electrophysiological synchrony and graph theoretical properties were associated with clinical and neuropsychological impairments. Finally, the epileptogenic pathology was the same (focal cortical dysplasia) for all children. Our main limitation is the absence of a control group, which has been previously reported for intracranial EEG [19].

The current report is the first to bridge the gap in knowledge between disturbances in functional connectivity caused by epilepsy and clinical impairments observed in affected children. We show that seizure-induced desychronization of Rolandic cortex is associated with contralateral motor hand deficits independently of the location of the SOZ. Furthermore, ictal disruptions in functional connectivity, shown by local declustering of the Rolandic cortex, are also associated with motor deficit following adjustment for SOZ location. Finally, our study has the advantage of correlating observed seizure-induced changes with clinical and neuropsychological outcomes, rather than statistical differences in BOLD signal on fMRI. These findings were highly significant in the gamma frequency range, and disturbance of network dynamics involving motor cortex were selectively related to motor function. We present evidence for a plausible mechanism for network impairment due to epileptic seizures.
